# Supplementary material for: Proteomic Identification of Pig Xenoantigens for Clinical Xenotransplantation
Source: bioRxiv. 2026 May 24:2026.05.22.727249. Preprint. [Version 1] doi: 10.64898/2026.05.22.727249 (PMC13228495; doi:10.64898/2026.05.22.727249)
Supplement: Supplement 1 — Supplementary Figures: Figure S1. The efficiency of antibody recovery. The bar graph illustrated the efficiency of antibody recovery using varying volumes of pig kidney protein (PKP) linked beads and human pooled plasma. The x-axis represents different ratios of PKP beads to plasma tested: 100 μl and 200 μl of PKP beads with plasma volumes of 250 μl, 500 μl, and 1000 μl. The y-axis shows the amounts of recovered antibodies. Figure S2. The distribution of fold changes in protein- and peptide-level enrichments. A. Histograms and density plots compared the distribution of fold changes in protein-level enrichments. Red Bar Plots: Fold change distribution of the experimental group (PK, beads with specific antibodies to pig kidney proteins) compared to their own average values, showing the data distribution. Blue Bar Plots: Fold change distribution of the PK group compared to the control group (E, beads with non-specific antibodies), indicating the enrichment significance. Purple Dashed Lines: Intersection of the two fitting curves used as the cutoff. B. Histograms and density plots compared the distribution of fold changes in peptide-level enrichments. Red Bar Plots: Fold change distribution of the experimental group (PK, beads with specific antibodies to pig kidney proteins) compared to their own average values, showing the data distribution. Blue Bar Plots: Fold change distribution of the PK group compared to the control group (E, beads with non-specific antibodies), indicating the enrichment significance. Purple Dashed Lines: Intersection of the two fitting curves used as the cutoff. [file media-1.pdf]

SUPPORTING INFORMATION FOR THE ARTICLE

Proteomic Identification of Pig Xenoantigens for Clinical Xenotransplantation

Hongyi Liu[1#], Trung Hoàng[1], Yingwei Hu[1], Yuanwei Xu[1], Zhenyu Sun[1], Brandon J. Peiffer[2], Yuanyu Huang[1] , Zhaoli Sun[2] , Hui Zhang[1*]*

1. Department of Pathology, Johns Hopkins University School of Medicine, Baltimore, MD 21231, USA

2. Department of Surgery, Johns Hopkins University School of Medicine, Baltimore, MD 21205, USA

* Corresponding author; Email: huizhang@jhu.edu (Hui Zhang), zsun2@jh.edu (Zhaoli Sun)

Table of contents

Figure S1. The efficiency of antibody recovery. S3
Figure S2. The distribution of fold changes in protein- and peptide-level enrichments.............S4

The efficiency of antibody recovery, related to Figure S1. (**Table S1**) (.xlsx)

The correlation results of protein and peptide-level enrichment. The data of the overlap of proteins enriched across three replicate samples (PK1, PK2, PK3) at the protein-level enrichment or peptide-level enrichment. The data of the overlap between proteins identified at the protein-level enrichment and proteins enriched at the peptide-level enrichment, related to Figure 2. (**Table S2**) (.xlsx)

Intersection analysis of protein and peptide-level enriched xenoantigens across cellular components and sequence alignment results, related to Figure 3. (**Table S3**) (.xlsx)

Glycopeptide analysis of the peptide-level enrichment, related to Figure 4. (**Table S4**) (.xlsx)

Figure S1

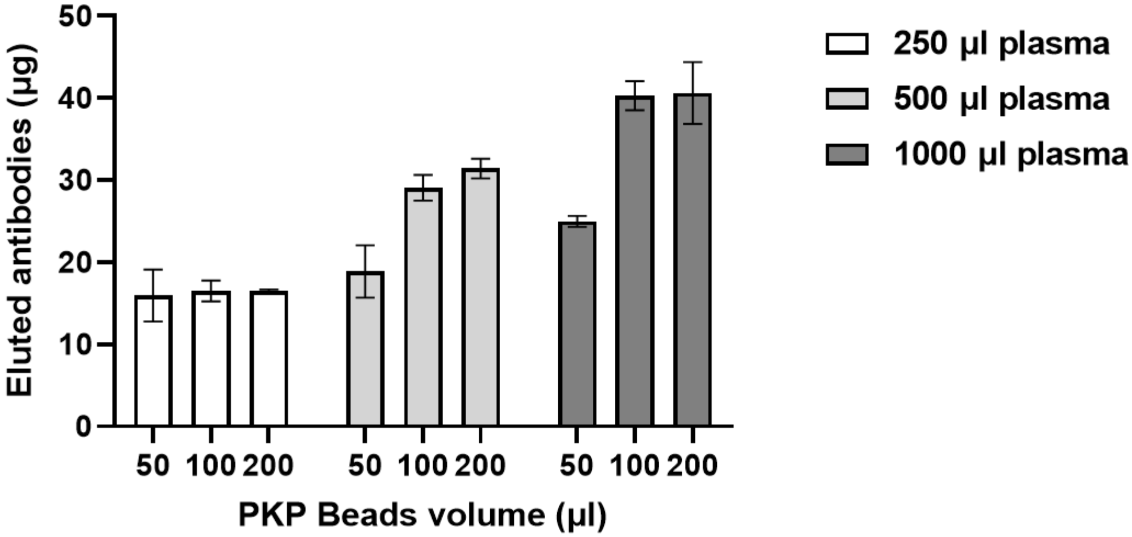

The bar graph illustrated the efficiency of antibody recovery using varying volumes of pig kidney protein (PKP) linked beads and human pooled plasma. The x-axis represents different ratios of PKP beads to plasma tested: 100 µl and 200 µl of PKP beads with plasma volumes of 250 µl, 500 µl, and 1000 µl. The y-axis shows the amounts of recovered antibodies.

Figure S2

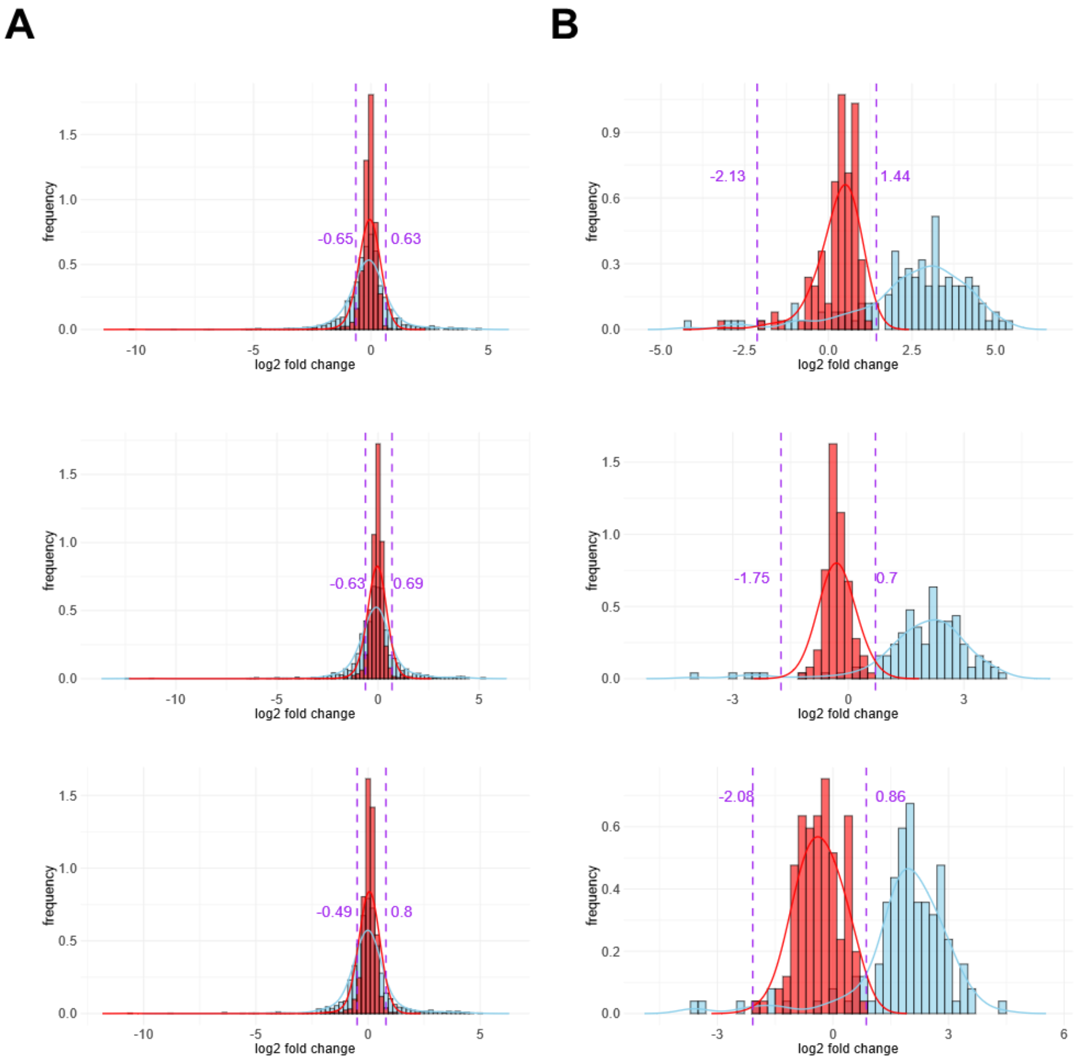

A. Histograms and density plots compared the distribution of fold changes in protein-level enrichments. Red Bar Plots: Fold change distribution of the experimental group (PK, beads with specific antibodies to pig kidney proteins) compared to their own average values, showing the data distribution. Blue Bar Plots: Fold change distribution of the PK group compared to the control group (E, beads with non-specific antibodies), indicating the enrichment significance. Purple Dashed Lines: Intersection of the two fitting curves used as the cutoff.
B. Histograms and density plots compared the distribution of fold changes in peptide-level enrichments. Red Bar Plots: Fold change distribution of the experimental group (PK, beads with specific antibodies to pig kidney proteins) compared to their own average values, showing the data distribution. Blue Bar Plots: Fold change distribution of the PK group compared to the control group (E, beads with non-specific antibodies), indicating the enrichment significance. Purple Dashed Lines: Intersection of the two fitting curves used as the cutoff.
